# HPV-Chlamydial Coinfection, Prevalence, and Association with Cervical Intraepithelial Lesions: A Pilot Study at Mbarara Regional Referral Hospital

**DOI:** 10.1155/2019/9092565

**Published:** 2019-01-10

**Authors:** Frank Ssedyabane, Diaz Anaya Amnia, Ronald Mayanja, Aisagbonhi Omonigho, Charles Ssuuna, Josephine Nambi Najjuma, Bwanga Freddie

**Affiliations:** ^1^Department of Medical Laboratory Science, Mbarara University of Science and Technology (MUST), P.O. Box 1410, Mbarara, Uganda; ^2^MMed OBGYN (MUST), Mbarara University of Science and Technology, Department of Obstetrics and Gynecology, P.O. Box 1410, Mbarara, Uganda; ^3^MMED PATH, Mbarara University of Science and Technology, Department of Pathology, P.O. Box 1410, Mbarara, Uganda; ^4^Msc (MUST), Mbarara University of Science and Technology, Department of Microbiology, P.O. Box 1410, Mbarara, Uganda; ^5^MNS (MUST), Mbarara University of Science and Technology, Department of Nursing, P.O. Box 1410, Mbarara, Uganda; ^6^PhD, (MUK), Makerere University, College of Health Science, P.O. Box 7072, Kampala, Uganda

## Abstract

**Background:**

Human Pappilloma Virus (HPV) is the necessary cause of cervical cancer. A number of risk factors are believed to influence the role of HPV in the development of cervical cancer. This is so because majority of HPV infections are cleared and only a few are able to result into cancer.* Chlamydia trachomatis *(CT) is considered a potential cofactor in the development of cervical intraepithelial neoplasia (CIN), although different studies have produced contradicting information (Silins* et al*., 2005, Bellaminutti* et al*., 2014, and Bhatla* et al*., 2013). The objective of this cross-sectional study was to determine the prevalence and association of HPV-Chlamydial coinfection with cervical intraepithelial lesions and other risk factors for cervical intraepithelial lesions at a hospital in south western Uganda (MRRH).

**Methods:**

The study included 93 participants, with an age range of 25 to 80 years, from whom cervical specimens were collected and enrolment forms were completed upon consent. Experienced midwives collected one cervical smear and two endocervical swabs from each participant. The swabs were used for HPV DNA and* Chlamydia trachomatis* antigen testing. Data was entered in Microsoft excel and analysed using STATA 12 software. With the help of spearman's correlation at the 0.05 level of significance, bivariate and multivariate analysis were done by logistic regression, to determine associations of risk factors to cervical lesions.

**Results:**

The results showed the prevalence of HPV-Chlamydial coinfection to be 8.6% (8/93). Positive Pap smear results were found in 60.22% (56/93) participants, most of whom had low grade squamous intraepitherial lesion (LSIL) (54.84%). HPV-Chlamydial coinfection showed a significant correlation with a positive cytology result and only relatively significantly correlated with LSIL grade of cytological positivity. HPV was found to be the risk factors associated with cervical intraepithelial lesions at MRRH.

**Conclusion:**

HPV, Chlamydia, and HPV-Chlamydial coinfection are prevalent infections and there is a likelihood of association between HPV-Chlamydial coinfection and with cervical intraepithelial lesions. This study recommends general sexually transimitted infections (STIS) screening for every woman that turns up for cervical cancer screening and a larger study, probably a multicentre study.

## 1. Introduction

Cervical cancer is the fourth most common cancer in women [1], and the seventh overall, with an estimated 528,000 new cases in 2012 [2]. A large majority (around 85%) of the global burden occurs in the less developed regions, where it accounts for almost 12% of all female cancers [2]. High-risk regions, with estimated age standardised rates (ASRs) over 30 per 100,000, include Eastern Africa (42.7), Melanesia (33.3), Southern (31.5), and Middle (30.6) Africa (WHO, 2013). Cervical cancer remains the most common cancer in women in Eastern and Middle Africa [2].

The World Health Organisation (WHO) reported an estimate of 266,000 deaths from cervical cancer worldwide in 2012, accounting for 7.5% of all female cancer deaths. It is further emphasized that almost nine out of ten (87%) cervical cancer deaths occur in the less developed regions. Mortality varies 18-fold between the different regions of the world, with rates ranging from less than 2 per 100,000 in Western Asia, Western Europe, and Australia/New Zealand to more than 20 per 100,000 in Melanesia (20.6), Middle (22.2), and Eastern (27.6) Africa [2].

The transformational changes start from a normal cell, turning it into a tumour cell in a multistage process, typically a progression from a precancerous lesion to malignant tumours. Because precancerous lesions can be detected by screening, vigorous screening programs have been put in place all over the world and have yielded results of reduced mortality in developed countries and a few developing countries [3, 4]. In Uganda, cervical cancer screening using visual inspection with acetic acid (VIA) and Pap smear has been in place for a good number of years, and of late, mass immunisation against high risk HPV (hrHPV) subtypes is encouraged for young girls all over the country [4, 5]. The Papanicolaou (Pap) cervical smear test introduced by George Papanicolaou in the 1920s is the most widely used cervical cancer screening method worldwide. However, limitations with respect to its sensitivity and specificity, compared with newer DNA methods, have prompted the search for alternative methods of identifying dysplastic squamous and glandular cells of the cervix. Further, research has been done on the aetiology of cervical cancer and a number of risk factors have been implicated in the acquisition and progression of the disease [6].

A number of sexually transmitted infections (STIs) have been linked to cervical cancer or precancerous lesions of the cervix. Some of these include HPV, HIV, and Chlamydia (Smith* et al.,* 2016). With increasing prevalence of HIV, there is a projected increase in risk to STIs in the developing world (Kakaire* et al*., 2015). Research studies conducted by Kakaire* et al*. (2015) showed a prevalence of STIs among women living with HIV to be 11.1% (95% CI 7.8-14.4) and individual prevalence for Trichomonas vaginalis, Neisseria gonorrhoea, and Chlamydia trachomatis being 5.9%, 5.4l%, and 0.9%, respectively.

STIs are a common health challenge in communities. It is estimated that 1 million sexually transmitted infections are acquired every day worldwide. Each year, there are estimated 357 million new infections with 1 of 4 common STIs (chlamydia, gonorrhoea, syphilis, and trichomoniasis), while more than 290 million women have an HPV infection (WHO, 2016). In view of this, Nwankwo and Magaji (2014) studied the prevalence of Chlamydia trachomatis in Nigeria and found out that the prevalence was 9.6% in Kano. They further noted the fact that a very high percentage of the patients, 95.2%, were completely ignorant of the existence of Chlamydia infection and consequently did not know of its associated complications.

*Chlamydia trachomatis *(CT) is considered a potential cofactor in the development of cervical intraepithelial neoplasia (CIN) although different studies have produced contradicting information [7–9]. It is further challenging to note that the vast majority of data about Chlamydia in cervical carcinogenesis is from the developed world. There is mixed epidemiologic evidence suggesting that HPV and* Chlamydia trachomatis* play a central role in the etiology of cervical intraepithelial neoplasia and subsequent cancer. It has also been noted that multiple non-HPV infections correlate significantly with low grade intraepithelial lesions (Vriend* et al.*, 2015). There are correlations not only between STIs and hrHPV infection but also between a positive cervical cytology and STIs (Kim* et al.*, 2016).

So, this study aimed at studying the relationship between Chlamydial infection, HPV infection, and HPV-Chlamydial coinfection and cervical intraepithelial lesions at MRRH.

## 2. Methods

### 2.1. Setting

The study was carried out in the cervical cancer clinic and the pathology department of Mbarara Regional Referral Hospital (MRRH) and Mbarara University of Science and Technology (MUST), respectively. MUST and MRRH are located in Mbarara district, south western Uganda. It is approximately 260 km from Kampala, the capital city of Uganda and 2 km from the town centre on the Mbarara-Kabale highway. MUST and MRRH are public institutions and the university has over 3000 students while the hospital is the regional referral hospital of south wersten Uganda with a capacity of 1200 beds. The cervical cancer clinic together with MUST clinical and research laboratory were chosen because they receive clients from the entire western region of Uganda; hence the sample size was representative of a big number of Ugandans.

### 2.2. Ethical Considerations

The principal investigator sought approval from the Department of Medical Laboratory Science, the faculty of medicine research committee and the Mbarara University Institutional Research and Ethics Committee** (Ref: MUREC 1/7).**

Clearance was also sought from the Department of Obstetrics and Gynecology before commencement of the study.

Respondents were given verbal explanation about the study and were given a consent form, well translated into Runyankole-Rukiga, (the local language,) where they signed, having known the relevance of the study.

Confidentiality was highly observed as respondents were identified with study numbers and not names.

## 3. Data Collection

The participants were recruited from the cervical cancer clinic in the months of June and July 2017. The study covered a total of 105 participants from whom samples were collected and then analysed. The study included all patients that presented at the cervical cancer clinic and consented to participate in the study. However all moribund patients and those that presented at the cervical cancer clinic during their menstrual periods were not considered for the study.

The study employed an enrolment form to collect qualitative data and laboratory methods. Three cervical specimens (one Pap smear and two endocervical swabs) were collected from every participant and labelled with unique study numbers.

Smears were made on glass slides, fixed immediately with 95% alcohol, and stained with the PAP staining method (Figure 2). Smears were examined by the principal investigator, together with a pathologist and a report kept. All the examination and grading followed the Bethesda grading system (2001). The remaining two endocervical swabs were used for* Chlamydia trachomatis* antigen testing and HPV DNA testing (Figure 3).* Chlamydia trachomatis* antigen testing was done immediately while one swab from every participant was stored at -20°C until shipment.

**Figure 1:**
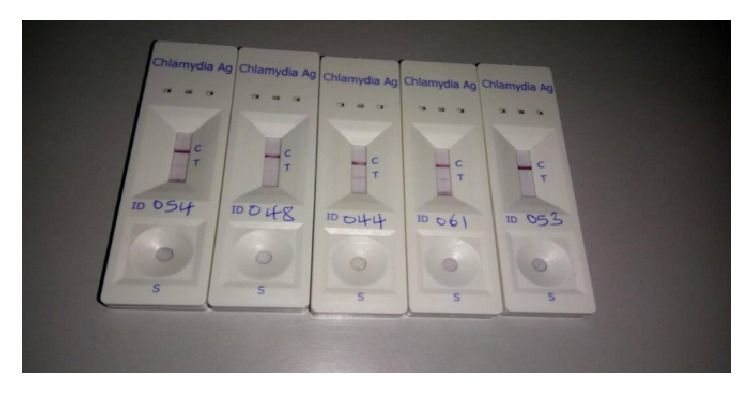
*Chlamydia trachomatis* antigen test.

A commercially available* Chlamydia trachomatis* antigen test kit - ce0123 (CTK BIOTECH INC) was used for qualitative detection of* Chlamydia trachomatis* DNA in cervical specimens (Figure 1). This kit had a sensitivity of 94.1% and a specificity of 97.4%. Positive and negative controls were purchased and run parallel with the samples, while following the manufacturer's instructions. Positive samples were confirmed with DNA PCR.

HPV DNA was extracted from endocervical swabs using the phenol chloroform method and then analysed using the classical Polymerase Chain Reaction (PCR) using the Amplisens HPV HCR genotype-Eph PCR kit. This Amplisens HPV HCR genotype-Eph PCR kit utilizes consensus primers, directed to a conserved L1 gene during amplification for hr HPV genotypes (16, 18, 31, 33, 35, 39, 45, 51, 52, 56, 58, and 59). In this multiplex PCR, the *β*-globin gene DNA was used as internal control plus other positive and negative controls. During the amplification 90°C, 60°C, and 72°C were set as denaturisation, annealing, and extension temperatures, respectively, for 40 cycles. After amplification, visualisation of the products was done by electrophoresis on a 1% agarose gel, stained with blue green loading dye.

Data from 93 participants was entered in Microsoft excel and analysed using STATA 12 software, with the help of spearman's correlation at the 0.05 level of significance. Bivariate and multivariate analysis were done by logistic regression, to determine associations of risk factors to cervical lesions.

## 4. Results

### 4.1. Population Characteristics

The study enrolled a total of 105 participants, of whom 12 were excluded from the study, because of unsatisfactory Pap smears. Eventually 93 participants were included in the data analysis. The mean age of participants was 41.92 years (minimum 26 and maximum 80), 55.91% of participants falling within the 25-40-year age bracket (Table 1).

### 4.2. Prevalence of HPV-Chlamydial Coinfection

HPV infection was found in 63.4% (59/93) of participants, whereas* Chlamydia trachomatis* infection was found in 8.6% (8/93) participants. And the HPV-Chlamydial coinfection was also in 8.6% (8/93) (Table 2).

For those women who were coinfected with HPV and Chlamydia, the mean age was 36 years of age, with a minimum of 29 and maximum of 52 years of age. Contraceptive use was reported in 2 out of 8 women (25%) whereas 25% of them reported to have a history of a positive Pap test. Smoking was reported in 1 out of 8 women (12.5%). HIV tests were negative in all the eight women who showed HPV-Chlamydial coinfection.

Bacterial vaginosis was reported in 88 out of 93 participants (94.6%). However, all women, 8 out of 8 (100%) who showed coinfection with HPV and Chlamydia, were positive for bacterial vaginosis.

### 4.3. Correlation of HPV-Chlamydial Coinfection and Other Risk Factors with Grades of Cervical Intraepithelial Lesions

**Figure 2:**
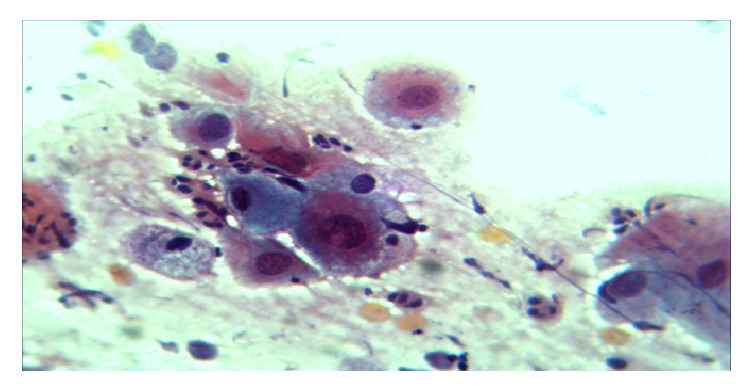
A photomicrograph of HSIL at X40 objective. Pap.

**Figure 3:**
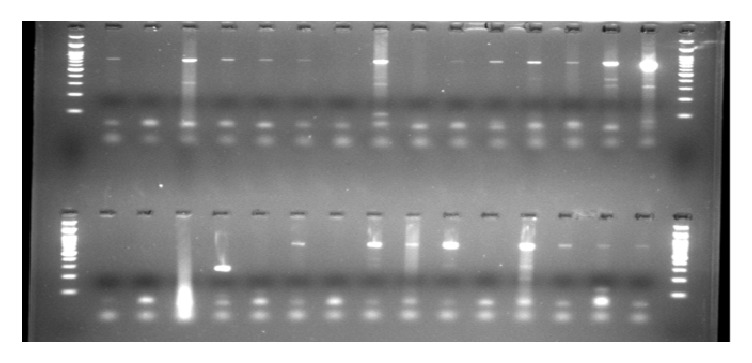
Gel1: Multiplex PCR gel for HPV types 16 and 18 and other HPV GTs: M=100bp ladder.

The eight women who had a positive* Chlamydia trachomatis* antigen test result, all of them had a positive Pap result (Figure 2). On further analysis all of the women with a positive* Chlamydia trachomatis* antigen test result had LSIL. HPV-Chlamydial coinfection showed a significant correlation with a positive cytology result (Spearman's rho = 0.2494, Prob > |t| = 0.0159) and significantly correlated only with LSIL grade of cytological lesion (Spearman's rho = 0.2784, Prob > |t| = 0.0069). ASCUS and HSIL had no significant correlation with HPV-Chlamydial coinfection (Spearman's rho = -0.0455, Prob > |t| = 0.6651 and Spearman's rho = -0.0560, Prob > |t| = 0.5938, respectively) (Table 3).

59 women tested positive for HPV DNA, and of them, 47 had LSIL, 2 had HSIL, and 1 had ASCUS, while 9 were negative for intraepithelial lesion or malignancy. Generally, HPV DNA positivity was significantly associated with Pap smear positive findings (OR 25.92593, 95% CI=8.359414 - 80.40679,* P value <0.001*), but specifically with LSIL (OR 29.375, 95% CI=8.665751 - 99.57482,* P value <0.001*) unlike other grades of lesions.

All eight women who had a positive Chlamydia antigen test result also had a positive HPV DNA test, which put the prevalence of HPV-Chlamydial coinfection at 8.6% (8/95). This could not be assessed on logistic regression because there were only 8 observations, which were below the required minimum (10).

Eight (8) risk factors were assessed for any association with cervical intraepithelial lesions. These included age, smoking, contraceptive use, HIV status, family history of cervical cancer, HPV positivity,* Chlamydia trachomatis* infection, and HPV-Chlamydial coinfection.

HIV infection, Chlamydia and HPV-Chlamydial coinfection had very few observations and therefore had insufficient data for logistic regression.

After controlling for other risk factors, HPV was still significantly associated with a positive Pap test results (LSIL) (OR: 39.26044, 95% CI= 8.145352 - 84.66311,* P value <0.001*) (Table 4).

## 5. Discussion

The prevalence of HPV-Chlamydial coinfection in this study was 8.6%, way above the reported rates of 0.7% as reported by Bhatla et al. [9]. Bhatla* et al*. [9] also reported lower prevalence of Chlamydial infection as well as HPV DNA positivity (4.8% and 18%, respectively). This difference could be due to the fact that our study enrolled some women who were below 30 years, who are believed to engage in risky sexual behaviour. Still, our study was conducted in a hospital setting, which brings in people with preexisting health complaints. It was comparable to the 7.2% and 13% got by de Castro-Sobrinho* et al*. [10] and de Paula* et al*. [11], respectively. However, da Silva Barros* et al*. [12] reported a higher prevalence of 27.4%, through their study, in which they used antibody based tests for Chlamydia.

This study found a significant association between HPV-Chlamydial coinfection and the cytological diagnosis of LSIL (Spearman's rho = 0.2784, Prob > |t| = 0.0069), unlike de Castro-Sobrinho* et al*. [10] who reported no correlation between Chlamydia infection or HPV-Chlamydial coinfection with cervical intraepithelial neoplasia. This disparity could be due to the fact that they recruited only HPV positive cases and also based on a positive Pap smear during recruitment

Studies by da Silva Barros* et al*. [12] confirmed an association between HPV-Chlamydial coinfection and cervical lesions. Specifically, they reported that HPV-Chlamydial coinfection was significantly associated with a diagnosis of high grade neoplasia. They found out that the prevalence of HPV was 86.3% and that of Chlamydia was 26%, while that of HPV-Chlamydial coinfection was 27.4%. In conclusion, they deliberated that the HPV-Chlamydial coinfection was significantly associated with HSIL (OR: 8.00, 95%CI: 1.31-62.62, P=0.007). This was in disagreement to ours, where HPV-Chlamydial coinfection was only significantly associated with LSIL (Spearman's rho = 0.2784, Prob > |t| = 0.0069), though we could not generate odds ratios. This disagreement could have been caused by the high prevalence of high grade lesions compared with what our study got.

This study investigated five risk factors for an LSIL. These included age (age brackets of 21 to 40, 41 to 60), HPV infection, Chlamydia infection, and HPV-Chlamydial coinfection. However, only HPV infection was found to be significantly associated with cervical intraepithelial lesions, specifically LSIL. This was additional information in terms of risk factors for cervical lesions. However, this was in disagreement with some authors who had earlier reported associations of some other sort. For example, many authors reported smoking, HIV, contraceptive use, and family history of cervical cancer as possible risk factors for cervical cancer. All these were found insignificantly associated with cervical intraepithelial lesions in our study.

For instance, Kjellberg* et al*. [13] conducted a study on risk factors to cervical neoplasia and found that HPV, smoking, and prolonged contraceptive use were significantly associated with higher grades of neoplasia. This was not the case in our study, in that smoking was discovered to be insignificantly associated with cervical lesions. This could be because of the few cases of smokers and lesions that were captured in this study. This could also be due to the fact that smoking is a nonprevalent habit in the study population.

In agreement with our findings, Bhatla* et al*. [9] conducted a pilot study and reported a high prevalence of HPV in the age group of 25-39 (69%), though the overall prevalence of HPV was 18%. Considering the risk of cervical cancer HPV poses to the individual, the age group of 21 to 40 qualifies as a risk factor.

Just like from our study, Castellsagué* et al*. [14] concluded that smoking was never associated with any grade of cervical lesions.

## 6. Conclusion

Based on the correlation results (Spearman's rho = 0.2784, Pro > |t| = 0.0069) there is a likelihood of an association between HPV-Chlamydial coinfection and LSIL. The only risk factor for cervical intraepithelial lesions that was confirmed was HPV infection.

## 7. Recommendations

From this study, we recommend routine STI screening for all women of child bearing age, that turned up for cervical cancer screening. This screening is also encouraged in mass screening programs as outreaches.

We also recommend a larger study, with greater power, that can gather information on more risk factors, which could have been confounders in this study.

## Figures and Tables

**Table 1:** Population characteristics.

**Variable**	**Frequency, n/93**	**Proportion,**%
**Age**		
25-40	52	55.9
41-60	32	34.4
61-80	9	9.7
**HIV status**		
Positive	2	2.2
Negative	91	97.8
**Smoking status**		
Smoker	4	4.3
Non smoker	89	95.7
**Family history of Cervical cancer**		
Yes	7	7.5
No	86	92.5
**Contraceptive Use**		
Yes	34	36.6
No	59	63.4

**Table 2:** Prevalence of Chlamydia, HPV, and HPV-Chlamydial coinfection.

**Variable **	**Frequency, n/93**	** Proportion, **%
**Chlamydia infection**		
Positive	8	8.6
Negative	85	91.4
**HPV infection**		
Positive	59	63.4
Negative	34	36.6
**HPV-Chlamydial coinfection**		
Positive	8	8.6
Negative	85	91.4

**Table 3:** Correlation of HPV-Chlamydial coinfection and other risk factors with grades of cervical intraepithelial lesions.

**Variable**	**Category**	**Positivity**	**ASCUS**	**LSIL**	**HSIL**
		Spearman's rho (Prob > |t|)	Spearman's rho (Prob > |t|)	Spearman's rho (Prob > |t|)	Spearman's rho (Prob > |t|)
**AGE**	25-40	0.0747 (0.4767)	-0.1670 (0.1097)	0.1516 (0.1469)	-0.0830 (0.4288)
	41-60	-0.1049 (0.3169)	0.0487 (0.6433)	-0.0704 (0.5024)	-0.1322 (0.2064)
	61-80	0.0431 (0.6813)	0.2022 (0.0519)	-0.1414 (0.1762)	0.3519 (0.0005)
**HIV**	Positive	0.1205 (0.2499)	-0.0220 (0.8344)	0.1345 (0.1985)	-0.0271 (0.7968)
**Smoking status**	Smokers	-0.0442 (0.6737)	-0.0314 (0.7649)	-0.1271 (0.2247)	0.2613 (0.0114)
**Family history ** **of Cervical cancer**	yes	0.1486 (0.1551)	-0.0423 (0.6873)	0.1770 (0.0896)	-0.0521 (0.6200)
**Contraceptive use**	Yes	-0.0672 (0.5222)	-0.1125 (0.2828)	0.0159 (0.8796)	-0.1386 (0.1852)
**HPV**	Positive	0.6602 (0.0000)*∗*	-0.0414 (0.6937)	0.6571 (0.0000)*∗*	0.0122 (0.9074)
**Chlamydia**	Positive	0.2494 (0.0159)*∗*	-0.0455 (0.6651)	0.2784 (0.0069)*∗*	-0.0560 (0.5938)
**HPV-Chlamydial ** **Coinfection**	Positive	0.2494 (0.0159)*∗*	-0.0455 (0.6651)	0.2784 (0.0069)*∗*	-0.0560 (0.5938)

*∗*P<0.05 (primary research data).

**Table 4:** Factors associated with positive Pap smear results, bivariate, and multivariate analysis.

**Variable**		**Bivariate analysis**		**Multivariate analysis**	
		**Unadjusted OR (95**%** CI)**	***P value***	**Adjusted OR (95**%** CI)**	***P value***
**Age**	**25-40**	1.36 (0.59-3.14)	0.472	0.065(0.006-0.73)	0.027
	**41-60**	0 .64 (0.27-1.52)	0.313	0.072(0.07-0.717)	0.025
	**61-80**	1.36 (0.32-5.81)	0.678	1.0	
**HIV**	**Positive**	1.0		1.0	
**Smoking**	**Smokers**	0.648 (0.09-4.82)	0.672	0.22(0.02-3.22)	0.269
**Contraceptives**	**Users**	0.75 (0.32-1.78)	0.517	0.99 (0.26-3.74)	0.987
**Family history of cervical cancer**	**Yes**	4.32 (0 .49-37.46)	0.184	0.90(0.85 -9.60)	0.931
**HPV**	**Positive**	25.93(8.36-80.41)	<0.001	39.08(9.35-163.58)	<0.001*∗*
**Chlamydia**	**Positive**	1.0		1.0	
**HPV-Chlamydial coinfection**	**Positive**	1.0		1.0	

*∗*P<0.05 (primary research data).

## Data Availability

The data used to support the findings of this study are available from the corresponding author upon request.

## Additional Points

*Significance of the Study*. This significance of this study was to provide scientific evidence on the current situation of HPV-Chlamydial coinfection and its relationship with Pap smear test results at Mbarara Region Regional Hospital (MRRH). The results were also hoped to guide decision-making on whether Chlamydia or even general STI screening should be added to the cervical cancer screening package for every woman attending the MRRH cervical cancer clinic.
